# Study Protocol: A randomized controlled trial of patient navigation-activation to reduce cancer health disparities

**DOI:** 10.1186/1471-2407-10-551

**Published:** 2010-10-13

**Authors:** Samantha Hendren, Jennifer J Griggs, Ronald M Epstein, Sharon Humiston, Sally Rousseau, Pascal Jean-Pierre, Jennifer Carroll, Amanat M Yosha, Starlene Loader, Kevin Fiscella

**Affiliations:** 1 University of Rochester Department of Family Medicine, Rochester, NY, USA; 2 University of Rochester Department of Community and Preventive Medicine, Rochester, NY, USA; 3 James P. Wilmot Cancer Center, Rochester, NY, USA; 4 University of Rochester Department of Psychiatry, Rochester, NY, USA; 5 University of Rochester Department of Emergency Medicine, Rochester, NY, USA; 6 University of Rochester Department of Pediatrics, Rochester, NY, USA; 7 University of Michigan Department of Medicine, Ann Arbor, MI, USA; 8 University of Michigan Department of Surgery, Ann Arbor, MI, USA

## Abstract

**Background:**

Cancer health disparities affecting low-income and minority patients are well documented. Root-causes are multifactorial, including diagnostic and treatment delays, social and financial barriers, and poor communication. Patient navigation and communication coaching (activation) are potential interventions to address disparities in cancer treatment. The purpose of this clinical trial is to test the effectiveness of an intervention combining patient navigation and activation to improve cancer treatment.

**Methods/Design:**

The Rochester Patient Navigation Research Program (PNRP) is a National Cancer Institute-sponsored, patient-level randomized trial (RCT) of patient navigation and activation, targeting newly-diagnosed breast and colorectal cancer patients in Rochester, NY. The goal of the program is to decrease cancer health disparities by addressing barriers to receipt of cancer care and promoting patient self-efficacy. The intervention uses trained, paraprofessional patient navigators recruited from the target community, and a detailed training and supervisory program. Recruited patients are randomly assigned to receive either usual care (except for baseline and follow-up questionnaires and interviews) or intervention. The intervention patients receive tailored assistance from their patient navigators, including phone calls, in-person meetings, and behind-the-scenes coordination of care. A total of 344 patients have been recruited. Outcomes measured at three month intervals include timeliness of care, patient adherence, patient satisfaction, quality of life, self-efficacy, health literacy, and cancer knowledge.

**Discussion:**

This unique intervention combining patient navigation and patient activation is designed to address the multifactorial problem of cancer health disparities. If successful, this study will affect the design and implementation of patient navigation programs.

**Trials Registration:**

clinicaltrials.gov identifier NCT00496678

## Background

Like the larger health care system in the United States (US), cancer care is fragmented, poorly coordinated and often not organized around the needs of the patient[1]. Many patients leave their health care visit confused about their diagnosis, prognosis, options for treatment, and next steps[2,3]. These problems are particularly severe for members of socially disadvantaged groups, including minority, low income, uninsured, non-English-speaking and low health literacy patients, and contribute to significantly higher cancer mortality rates[4].

**Figure 1:**
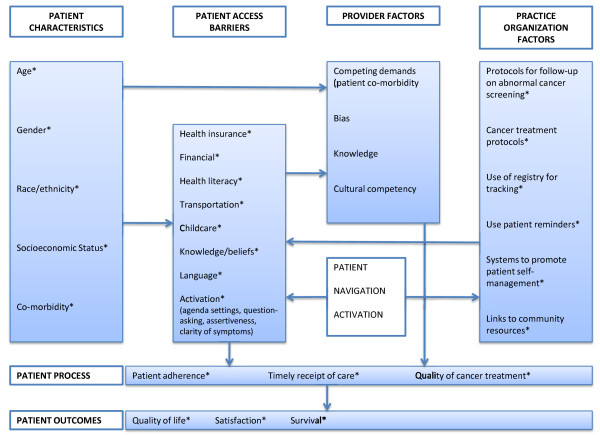
**Theoretical Model Linking Patient, Provider and Health System Factors to Cancer Health Disparities**[41]. (*indicates factor measured in the present study)

Healthcare disparities based on social disadvantage, including race, ethnicity, socioeconomic status, language, and insurance, have been well documented for cancer screening[5], follow-up of abnormal cancer test results[6,7], and cancer treatment[7,8], [9]. The causes for disparities are multifactorial, including patient characteristics such as low health literacy, patient access barriers such as lack of healthcare insurance, provider factors such as cultural competency, and practice organizational factors such as a lack of systems to track patients at risk (Figure 1). In addition, interactions between factors may play an important role, particularly in the case of poor clinician-patient communication[10-12]. Studies in which equal treatment is meticulously provided to patients, irrespective of race, ethnicity, and socio-demographic backgrounds, have shown a significant reduction in disparities in treatment outcome for patients with breast and colorectal cancer,[13-15] indicating the potential to decrease cancer health disparities.

Two different models to address these problems have emerged in recent years: patient navigation (PN) and patient activation (PA) often referred to as "patient empowerment". We briefly review the history of each, discuss a conceptual framework for integration of the two into the same model, and conclude with our experience in implementing the integrated model in the setting of an ongoing randomized controlled trial.

### Conceptual Framework for Navigation-Activation

Because the causes of cancer health disparities are multi-factorial, an optimal intervention to address disparities must address different dimensions of the problem. The navigation-activation program combines PN and PA to improve patient-physician communication. These components are described briefly and the combined program is diagrammatically presented.

### Patient Navigation

For more than 40 years, community health workers (CHW's) have enhanced access to care among underserved populations in the United States (US)[16,17]. Freeman *et al *reported that 87% of inner-city patients with abnormal cancer findings who received PN from CHW's completed recommended biopsies compared to only 57% among those who were not navigated[18]. More recently, CHW's have been shown to improve outcomes in prenatal care[19], smoking cessation[20], child[21] and adult immunizations[22], and diabetes and depression management[23].

Patient navigation programs for cancer-related care have been developed across the US, including more than 100 PN programs sponsored by the American Cancer Society. Most PN programs share a number of common characteristics. Navigators assist patients with abnormal cancer screening or diagnostic tests by identifying and addressing barriers to quality and timely health care. For example, PN's may help patients keep track of appointments, interpret medical information, and provide social support[24].

### Patient Activation

Randomized controlled trials have demonstrated that interventions to train patients to assume more active involvement in their care and to ask more appropriate and relevant questions ("activation") can improve patients' outcomes[25,26]. Activation improves patient satisfaction with care and physician counseling[27,28], and adherence to treatment and follow-up appointments[29]. Pre-visit planning and coaching of breast cancer patients improves both patient and physician visit satisfaction[30]. Patient question-asking and provision of clear information by clinicians is associated with fewer diagnostic delays among black women with abnormal mammograms[12]. Patient assertiveness may attenuate racial and socioeconomic disparities in breast cancer evaluation[31]. These findings suggest that "activating" patients may be one means of improving cancer care for disadvantaged patients. However, not all such studies have positive results, and the specific techniques and settings of activation interventions are probably important to achieving desired results[32,28].

In prior studies, "activation" has been conceptualized as an intervention of limited duration, intended to provide education and empowerment. Conversely, PN has been structured as an ongoing, longer-term relationship between the patient and the navigator that provides a variety of supports. Navigation-activation brings activation together with PN as a longitudinal intervention. How can this intervention potentially decrease health disparities and improve health outcomes? Figure 1 provides a theoretical model to explain how this intervention may improve outcomes by affecting factors that mediate cancer health disparities. Disparities in care related to social disadvantage are mediated through *patient *access barriers, *provider *factors, and *practice *organizational factors. By potentially reducing delays in patient care and improving patient adherence and quality of care, patient navigation-activation seeks to improve quality of life, satisfaction, and survival.

### Cancer Health Communication

Effective communication between patients and healthcare providers is critically important for optimal care[33]. Poor communication may undermine patients' ability to understand their treatment options, cope with anxiety caused by cancer, make informed decisions regarding diagnostic and therapeutic procedures[34-36], and adhere to treatment[37].

The navigation-activation model is consistent with a recently-proposed conceptual framework for developing evidence-based interventions to improve health care communication[38,39]. A recent National Cancer Institute report on patient-centered communication suggests pathways by which effective communication is linked to high-quality medical decisions, adherence, patient and clinician satisfaction and improved health[38,40]. Navigators directly address *patient self-management*, one of the essential functions of communication. Through forming strong therapeutic relationships and providing both emotional and instrumental support, navigators assist patients in information exchange with providers, as well as other essential communication functions.

Navigators can help patients forge a more participatory dialogue with their clinicians. Specifically, navigators help patients ask clinicians questions that lead to a clearer understanding of their illness and treatment. They also coach patients to better inform clinicians about their experiences and preferences. Through this improved dialogue, patients come to posses both the information and support to participate effectively in decisions regarding their health.

## Methods/Design

### Study Design

The University of Rochester Patient Navigation Research Program (UR-PNRP) is a National Cancer Institute-funded, 5-year, randomized trial of PN for patients with a new diagnosis of breast or colorectal cancer, or with a positive screening test for these diseases[41]. The primary outcomes of the UR-PNRP are timeliness of care, guideline-concordant care, patient satisfaction, and quality of life.

### Selection of Study Sites

All oncology practices in the city of Rochester were recruited to participate in the PNRP program, as well as several primary care practices in Rochester, and several oncology practices in the surrounding community.

### Patient eligibility, recruitment, consent and randomization

**Figure 2:**
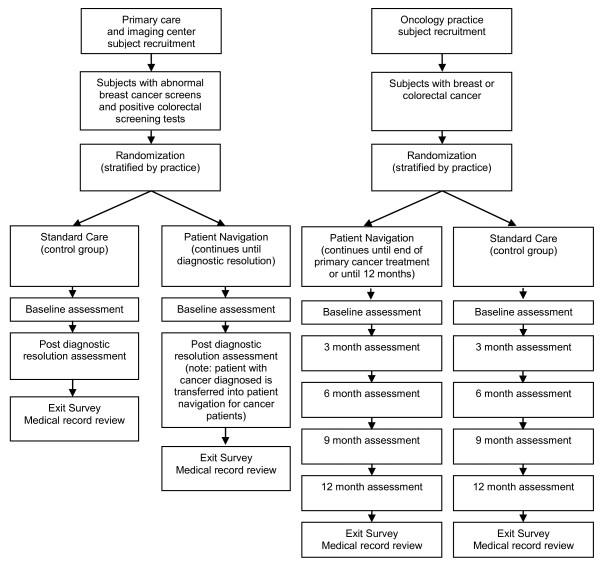
**Diagram of Patient Flow through the Clinical Trial**.

Patients eligible for participation in the PNRP study are newly-diagnosed breast and colorectal cancer patients from participating practices, and patients with a positive screening test for these diseases. There are no exclusion criteria based upon socioeconomic status, race or insurance, but institutionalized patients and those with dementia or prior cancer (other than non-melanoma skin cancer) are excluded. Figure 2 is a flow chart of patient enrollment, randomization and progress through the trial. All study procedures have been approved by the Institutional Review Boards of the University of Rochester and participating sites in the area. All patients participating in the study provide written informed consent.

### Data Collection

Patient information is collected by research assistants prior to randomization. Patient demographic information is obtained from medical records and patient self-report, via in-person interview. Outcome measures are collected for both intervention and control patients at baseline and at three month intervals, as detailed in Table 1. In addition, information on barriers to care was collected by patient navigators, in the intervention group only, during semi-structured interviews with patients. A standardized form was used. Navigators also keep detailed records of their activities performed on behalf of their caseload of navigated patients, and the time each activity took.

**Table 1:** Outcome and Other Measures of the Patient Navigation Research Program

Category	Title	Collection Times (mo)
**Primary Outcome Measures**		
Time to completion of Treatment	Time to completion of Treatment	
Satisfaction	Patient Satisfaction with Care	0;3;6;9;12
Cost	Cost	
		
**Knowledge/Health Literacy**		
Health literacy	Rapid Estimate of Adult Literacy in Medicine (REALM)	0
Knowledge	Breast Cancer Treatment Knowledge	0;3;FU
Knowledge	Colorectal Cancer Treatment Knowledge	0;3;FU
		
**Quality of Life**		
Quality of Life	Impact of Events Scale (IES)	0;3;6;9;12
Cancer Related Health Status	Functional Assessment of Cancer Therapy-Breast Cancer (FACT-B)	0;3;6;9;12
Cancer Related Health Status	Functional Assessment of Cancer Therapy-Colorectal Cancer (FACT-C)	0;3;6;9;12
		
**Medical Conditions**	Charlson Commorbidity	chart review
		
**Adherence**		
Adherence	Medical Outcomes Study (MOS) General Adherence Items	0;3;6;9;12
Beliefs	Beliefs about Medication Questionnaire (BMQ)	0;3;6;9;12
		
**Interaction**		
Attachment Style	Relationship Questionnaire (Attachment Style)	0
Intensity	Navigator Perceived Time and Emotional Intensity of Patient Navigation	0;3;6;9;12
Self Efficacy	The Communication and Attitudinal Self-Efficacy Scale (CASE)	0;3;6;9;12
Satisfaction	Patient Satisfaction with Navigator	3; FU
Tracking	Patient Navigator Tracking Logs - encounter times, barriers, and actions	Throughout

### Recruiting and Training Patient Navigators

Non-medically-trained persons from the community were recruited to be PN's. Minimal selection criteria included a high school degree[42], reliable mode of transportation and a current driver's license. Preference was given to applicants with experience in case management. Desirable personal qualities included strong interpersonal and communication skills, fluency in Spanish and English, ability to learn, dependability, initiative, and passion and commitment to improving health care for underserved patients.

### The Navigator Curriculum

The first navigators received a six-month, intensive training program that covered the basics of cancer diagnosis and treatment and each of the PN tasks, as outlined in Table 2. Subsequently, the training has been shortened to less than half this time. All navigators also completed an 80-hour core curriculum (The Cornell Project) developed by New York State for the training of CHW's and yearly two to three day national training seminars, sponsored by the American Cancer Society. Project investigators, other medical professionals, local government resources, and local private agencies were recruited to participate in the training program. Training in patient activation involves a series of observed and evaluated interactions with trained, standardized cancer patients (actors trained to portray patients). A navigator manual was created, including standard operating procedures, lists of community resources and patient educational materials, as well as materials to reinforce the training curriculum.

**Table 2:** Navigator Training Program

Training Activity	Concepts	Educator(s)
**In-House Training**		

Cancer Health Disparities Overview	Patient, provider, practice and health system barriers to care	Program director
Research 101	Human subject protection, HIPAA^a^	Project coordinator
Standard Operating Procedures and Case Management		Project coordinator, PN supervisor
Orientation to Participating Health Care Settings	Visit practices; computer medical database training	Project coordinator, PN supervisor
Cancer 101	Physicians teach PN's basics of cancer diagnosis and treatment; shadowing MD's at patient office visits, colonoscopies; observing tumor board conference at cancer center; visit to pathology laboratory	Medical oncologist, surgical oncologist
Interpersonal Communication 101	"How to talk to your doctor", "Patient empowerment", others	PN Supervisor; communications researcher; staff of non-profit agency
Computer Literacy	Email; Microsoft office; computerized calendars; internet searches for resources and patient care guidelines	Project coordinator, research assistants
Domestic Violence	Child protective services and domestic violence lectures	Department of Health and Human Services
Community Resources	Transportation, financial counseling, interpreter services, child care, social work	Social worker from cancer center
Motivational Interviewing	Self-determination and empowerment	Research assistant
End of Life Care	Transition from treatment to palliation	Hospice nurse
Personal Safety	Actions to minimize risk	Police department
The Health System	Review of government and private health insurances	PN supervisor
The Initial Assessment Interview	A semi-structured interview was designed, and PN's learned to conduct it, using the skills of "joining", history-taking, assessment of patient barriers, and making follow-up plans	PN supervisor

**External Training**		

Cornell Empowering Families Project	New York State program for CHWs. Topics include: relationship and communication skills; cultural competence; working with low-literacy patients; needs assessment; empowerment; CHW self-care	The Cornell Project
Simulated Patient Training	Simulated patients, with video-taping and feedback	Local, private agency that provides standardized patients for medical training
NCI/ACS^a ^Navigator Training Summit	4 day program with navigators from across the US	

^a^HIPAA = Health Insurance Portability and Accountability Act; NCI = National Cancer Institute; ACS = American Cancer Society

All of the navigators completed the training program prior to working with patients. The success of navigator training was evaluated in several ways. The core CHW curriculum (The Cornell Project) required post-testing to obtain certification. In addition, PN's were regularly observed by their supervisor during patient encounters. Encounter forms were checked for accuracy and consistency. Computer skills were supervised to ensure minimal standards were met. Passing scores on tests for research ethics certification and patient confidentiality were required. PN's were also pre- and post-tested at national training sessions.

### Characteristics of Navigators

The first three PN's recruited were females: one African-American and two Puerto Rican-Americans who were fluent in Spanish. Over time, three additional Navigators have been hired and trained, including two African-American females and one white male. None of the PN's had any previous medical training; however, PN's had previous experience with health insurance enrollment, being a patient in the medical system, and/or previous community work experience.

### Supervision for Navigators

Navigators are supervised by a master's level social worker with experience in clinical supervision, teaching, research, and work in primary care. The navigator supervisor provides real-time consultation and supervision of case management, through weekly meetings with each of the navigators, cell phone contact, review of documentation, and review of selected audiotapes (with patients' consent) of meetings between navigators and patients. Supervision also includes professional support of the PN's, recognizing the emotionally intense work they are doing and promoting self-care to minimize "burnout". Quarterly, each PN is directly observed by the supervisor in patient interactions to ensure that basic competencies continue to be met.

### Description of the Navigation Intervention

After enrollment and completion of baseline data collection, patients are randomly assigned to standard care or patient navigation-activation. Patients assigned to navigation are contacted within three days by their PN to assess their needs. PN's conduct a semi-structured assessment interview in-person or over the phone to identify barriers to care. Table 3 lists the types of barriers that PN's were trained to address. Navigators also immediately become involved in performing proactive services on the patient's behalf, such as appointment reminders, in order to prevent delays in receiving treatment or follow-up care with their medical provider.

**Table 3:** The Navigation-Activation Intervention

Phase 1: Patient Intake	Identify Barriers to Quality Care
	Transportation
	Housing
	Insurance
	Literacy
	Language
	Child Care/elder care
	Family/Community Supports
	Geographic Distance from Health Care
	Adequate Health Insurance
	Financial Problems
	Work Schedule
	Medical Communication Concerns
	Fear
	Comorbid Illnesses
	Disability
	Perceptions/Beliefs about Treatment
	Systems Problems with Scheduling Care
	Attitudes toward Providers
	Other
**Phase 2: Patient Navigation-Activation**	**Address Barriers**
	Provide Information
	Provide Pre-Appointment Coaching
	Provide Emotional and Social Support
	Provide Appointment Reminders
	Link to Resources

**Phase 3: Assessment of Intervention**	**Outcomes**
	Time to Completion of Treatment
	Treatment Guideline-Compliant Care
	Patient Satisfaction
	Patient Knowledge and Self-Efficacy

After identifying the patients' barriers to care, the PN uses a variety of strategies to address the barriers. Actions taken by the PN include: supportive contact with the patient, such as face-to-face meetings, telephone, email or regular mail correspondence; identifying and linking patients to social or financial resources and appropriate community supports; helping with paperwork, obtaining records, scheduling appointments, following-up on test scheduling or results; and accompanying the patient to appointments to help coach as well as providing emotional support.

Navigators provide patients with approved educational materials and promote treatment adherence. Navigators facilitate coordination of care by ensuring that consultation reports, test results, and new prescriptions or prescribed treatments are available to all providers at the time of an appointment. Navigators notify providers when a patient misses an appointment or treatment or experiences new or changed symptoms. Navigators are specifically trained to effectively interact with professional health care personnel.

For patient activation, PN's offer all patients coaching in communication skills. Navigators meet with patients prior to appointments and assist patients in identifying one to three concerns they wish to address during their visit, and create question lists as tools for patients to use during visits. Patients rehearse how to ask questions, follow-up when they are unclear about the response they receive, and re-state the plan to confirm understanding. Though often present during visits, PN's encourage patients to assume an active role during visits, rather than speaking for them. Navigators debrief with patients following the medical visits, to identify and clarify potential areas of confusion.

### Tailored Intensity of Navigation

Participating patients receive navigation of differing intensity depending on their access barriers, needs, and resources. Patients who report few or no access barriers and report no need for navigation services receive *low-intensity navigation*. For example, a woman with an abnormal mammogram who requires no assistance and does not desire coaching in communication receives support and reminder phone calls only. *High intensity navigation *is indicated for a patient with cancer who faces access barriers to care or who experiences significant complications from treatment requiring intensive coordination of care among providers.

In summary, the navigation-activation intervention is designed to identify barriers, assist patients in overcoming them, provide emotional support, improve patient understanding of his or her condition and treatment, empower the patient to become actively involved in his or her care, improve adherence to treatment, and promote patient satisfaction with health care. Thus, the intensity of navigation is tailored to the needs of the patient.

### Patient Accrual and Study Flow

The clinical trial began in 2005 with a planning and development phase followed by recruitment of three CHW navigators and a supervisor in 2006. Three hundred forty-four patients have been recruited into the program, and 178 were randomly assigned to the navigation-activation intervention group, following informed consent. In addition to the planned assessments, semi-structured exit interviews are also being conducted with all subjects as they complete the program to assess their cancer-treatment experiences and their experiences with the navigators, for quality control.

While the study is still on-going, we can report that common barriers identified and addressed by the patient navigators include financial difficulties, lack of health insurance, lack of social support, lack of transportation, difficulty communicating with health care providers, and limited English proficiency. In addition to planned help provided by our navigators to address these barriers, navigators also help patients organize and follow-through with doctors' appointments, and helping them understand medical information.

### Planned Analytic Approach

The primary outcome measures for this clinical trial include time to completion of treatment (or to diagnostic resolution in the case of patients with positive screening tests), patient satisfaction, and cost. Details regarding each of these measures are as follows:

#### Time to completion of cancer treatment

This measure is defined based on review of medical records following completion of treatment for oncology subjects. It will be assessed based on time (in days) from the date of the initial oncology/surgical consultation to completion of cancer treatment (e.g. surgery, chemotherapy regimen, or radiation, whichever occurs last).

#### Health care satisfaction

We hypothesize improvements in satisfaction with health care. Baseline satisfaction is measured using a newly created patient satisfaction with cancer care (continuous measure). It is assessed at baseline for all subjects and at three months for primary care subjects and at three months, six, nine and twelve months for subjects with cancer or at the end of treatment.

#### Costs of navigation

We assess the costs of navigation including supervision. We assess the costs of navigator time by asking navigators to track their time spent on navigaton-related functions. These include training time and in-services, meetings with supervision, entry of data for subjects assigned to navigation, phone calls related to navigation, meetings with subjects and families, meetings and phone calls with providers and office staff, and travel time. The proportion of time devoted to navigation will be multiplied by navigator cost (salary plus benefits). Additional expenses include costs for supervision, transportation, child care, and patient educational materials. Patient navigator supervision time will be based on the percent of time related to navigation. We also collect data (e.g. medical records) needed to estimate the health care costs of subjects assigned to either navigation or usual care. Subjects are also asked to estimate time (nearest day) that they missed work to visits or disability.

Secondary outcome measures for this study are guideline concordant care, patient adherence, health literacy, patient activation, medical knowledge, and functional health status. Details regarding analysis of these outcomes include:

#### Guideline concordant cancer care

This is a dichotomous measure, defined as concordance (yes/no) between diagnostic and therapeutic management documented in the medical records, and national guidelines for evaluation and treatment of breast or colorectal cancer according to stage, age, and hormone receptor status. An oncologist (JG) will assess guideline concordant care by reviewing abstracted data, and will be blinded to group assignment. Depending on the findings, use of an ordinal measure will also be considered.

#### Patient adherence to recommendations

We will measure patient adherence from both chart abstraction and patient report. Patient adherence to diagnostic recommendations is based on the percentage of missed appointments for diagnostic testing. Subject adherence to treatment is assessed based on the percent of recommended courses of radiation received or percentage of full chemotherapy received and percentage of cancer visits that are missed. We also are measuring relative dose intensity (RDI) for breast cancer patients who receive chemotherapy. RDI is a standard measure of optimal chemotherapy dosing. Dose intensity is amount of drug delivered per unit time typically standardized to body surface area as mg/m^2^/week. The RDI is the ratio of the dose intensity actually received relative to optimal dose intensity.

#### Health literacy

We hypothesize that persons with low health literacy will derive more benefit from navigation than those with higher literacy. The Rapid Estimate of Adult Literacy in Medicine (REALM) for English speakers and the Short Assessment of Health Literacy for Spanish Adults (SAHLSA) will be used to assess health literacy among all subjects at baseline. Less than seventh grade reading level will be considered low-literacy. Secondary analyses will examine health literacy as a continuous variable. This measure is omitted among the few subjects who do not speak English or Spanish.

#### Self-Efficacy

We assess patient self-efficacy among all subjects using the Communication and Attitudinal Self-Efficacy Scale for cancer (CASE)[43]. CASE scores are assessed at baseline, three months, six months, and twelve months for subjects with cancer.

#### Knowledge

We anticipate that navigation will be associated with improvements in knowledge. We will assess knowledge about breast cancer and colorectal cancer and its treatment using instruments developed by our investigators. It will be assessed at baseline for all subjects and at three months for primary care subjects and at six and twelve months for subjects with cancer.

#### Functional health status

We will assess overall quality of life using the Functional Assessment of Cancer Therapy[44] scales (FACT-C and FACT-B). These will be assessed at baseline among all subjects, at three months among primary care subjects and at six and twelve months among subjects with cancer.

### Sample Size Determination

Sample size calculations were calculated for time to completion of treatment for diagnosed cancer patients, specifically to assess differences between the intervention and control groups in the proportion of patients who complete treatment at nine months. We estimate that 75% of control subjects will have completed cancer treatment at 9 months. Based on our original projection of 200 enrolled cancer patients, we have 80% power to detect a difference in treatment completion rates of as little as 16% between the navigated and standard care patients. With 300 enrolled cancer patients, this detectable difference improves to 13%. These detectable effect sizes are clinically meaningful. These estimates also apply to any of the outcomes involving proportions such as rates of patient adherence or receipt of guideline concordant care.

### Data Analysis

Continuous measures will be compared between randomized groups, using two-sample *t*-tests. These measures include times, mean satisfaction and quality of life scores. Effect sizes are expressed in terms of standard deviations of the measure given that we have no a priori estimates of the distribution parameters. With the noted sample sizes for cancer treatment subjects, the expected sample will provide enough power to detect differences in mean measures of as little as 0.4 standard deviations. Cohen suggests that effect sizes of 0.2, 0.5 and 0.8 standard deviations can be considered "small", "moderate" and "large". We expect to be able to detect moderately-small effects with this analysis and should have power to detect clinically meaningful differences between the groups.

At the conclusion of the trial, analysis will include local as well as multi-institutional outcomes comparisons between intervention and control patients, combining data from multiple institutions performing trials of patient navigation funded by the National Cancer Institute, under the multi-institutional granting structure.

## Discussion

The elimination of health disparities is one of two overarching goals for Healthy People 2010[45]. Eliminating disparities in cancer treatment has to date defied solution[8]. Patient navigation is among the few promising methods for doing so. However, most PN programs largely focus on assisting patients with logistical barriers and improving care coordination. In this paper, we describe integration of this "traditional" PN with patient activation through coaching. We outlined the steps we have taken to implement the program as part of NCI's Patient Navigation Research Program.

Numerous patient navigation programs are currently funded throughout the US, or in planning stages, often with little theoretical or practical guidance on which to base their designs and implementation. As cancer centers, foundations, and others throughout the US seek to implement PN programs, the experiences described in the present study will be valuable. The conceptual framework and training structures presented here are also relevant to other medical conditions such as HIV, chronic renal disease, or chronic pain among others, that might benefit from PN.

During the design and implementation phase of this trial, unanticipated challenges arose, requiring flexibility to address them. First, the program was initially designed to receive most referrals from the primary care setting for abnormal screening follow-up. However, due to changes in radiology centers' protocols to more directly handle abnormal screening follow-up themselves, navigation for screening was no longer needed in our system. We therefore shifted PN activities to focus on cancer patients, and redirected recruitment to the community's cancer centers.

Another key challenge has been learning how to effectively navigate very high need patients. When patients' financial and social needs exceeded what the program could realistically provide, navigators learned to set limits, particularly when patient requests went beyond helping with cancer-care-related needs. Another challenge has been in recruitment and retention of stable and highly motivated CHW's for patient navigation. Two PNs have left the program and recruitment and training can be time consuming. Our group has concluded that close supervision of patient navigators with group discussion of cases is the key to providing effective support for PNs.

The *patient activation *component of this intervention is unique and is also one of the more difficult tasks confronting the PNs, who seek to empower patients without straining the relationship with cancer care providers. The extensive training program has equipped them to tackle this challenge, and patient and informal provider feedback has been positive. The formal evaluation of patient satisfaction and quality of life will occur at the end of the study, along with measurements of guideline-concordant care and time to completion of care. We hypothesize that these parameters will be improved compared to patients receiving standard care.

Despite the widespread implementation of PN programs in the US, objective success of PN in reducing cancer health disparities remains unproven[46]. The program described here and the overall NCI-PNRP initiative seeks to answer the question: will the investment in PN pay dividends in measurable patient outcomes? The results of our randomized, controlled trial will attempt to show if care quality, timeliness and satisfaction are improved by this intervention, and whether racial or ethnic disparities in these measures are reduced.

In conclusion, patient navigation and patient activation *can be combined *into a single intervention to promote optimal cancer care for underserved patients. Carefully selected, non-medically-trained CHW's can be trained to assist patients and coach them to communicate more effectively with their physicians, although close supervision is essential. If results of the randomized trial indicate a benefit of this combined intervention on timeliness, guideline concordance, patient satisfaction, and/or other important outcomes, this program can serve as a model for future PN program design.

## Competing interests

The authors declare that they have no competing interests.

## Authors' contributions

KF and JG designed the clinical trial, obtained funding and critically revised the manuscript. SH (Hendren) provided cancer clinical expertise and drafted the manuscript. RME provided expertise on cancer communications and critically revised the manuscript. SH (Humiston) provided expertise on patient outreach and critically revised the manuscript. SR provided expertise on navigator training and supervision, and critically revised the manuscript. PJP provided expertise on development of outcome assessment measures and critically revised the manuscript. JC, AY, and SL provided accurate descriptions of the navigator training program and intervention, and critically revised the manuscript. All authors read and approved the final version of the manuscript

## Pre-publication history

The pre-publication history for this paper can be accessed here:

http://www.biomedcentral.com/1471-2407/10/551/prepub
